# Improving Documentation of Bowel Movements Using the Bristol Stool Chart: A Quality Improvement Project in a District General Hospital in the United Kingdom

**DOI:** 10.7759/cureus.100902

**Published:** 2026-01-06

**Authors:** Sahar Yaseen, Fatema Kamaleldien Mohamed Abuelass

**Affiliations:** 1 Internal Medicine, Royal Blackburn Teaching Hospital, Blackburn, GBR; 2 Renal Medicine, Nottingham University Hospitals NHS Trust, Nottingham, GBR

**Keywords:** bowel habits, bristol stool chart, clinical documentation audit, clinical documentation improvement, inpatient care, prevent complications, quality improvement project (qip)

## Abstract

Background

Patient-centred care is a fundamental aspect of the NHS. As healthcare professionals, we tailor the care plan to each patient’s individual needs, ensuring a patient-centred approach.

An often overlooked aspect of this care plan is the documentation of bowel movements. This is a vital part of patient care, whether patients are elderly or young and independent in their activities of daily living (ADLs).

Failure to document bowel movements can lead to misdiagnosis and worsening of conditions such as constipation and diarrhoea. This may potentially prolong hospital admission.

The Bristol Stool Chart is a valuable guide for healthcare professionals in differentiating stool types. Correct and consistent documentation allows early intervention, whether that involves prescribing or stopping laxatives, sending a stool sample for culture and sensitivity, or placing the patient in a side room to prevent the spread of infection.

Aim

This Quality Improvement Project (QIP) aims to enhance patient care by improving the documentation of bowel movements using the Bristol Stool Chart in patients admitted to the hospital. The aim is to assess the quality and consistency of bowel movement documentation in 30 patients on the Endocrine and Diabetes ward; determine compliance with documentation standards; identify gaps in recordings; identify reasons why bowel movements are not recorded; and recommend improvements.

The primary aim of this QIP was to enhance patient care by enabling prompt and appropriate treatment, preventing deterioration, and avoiding unnecessary prolonged hospital admission due to unresolved bowel issues.

Methodology

This is a retrospective study that involved 30 patients admitted to the Endocrine and Diabetes ward in a District General Hospital. Patients were selected randomly, independent of age and sex, from the electronic patient records. The QIP was designed using a Plan-Do-Study-Act cycle and was conducted over a three-month period. Patients were selected using a set of inclusion and exclusion criteria. We included patients who had a hospital stay greater than 3 days and excluded patients who were on the end-of-life (EOL) pathway or had incomplete documentation due to transfer or early discharge. Data were collected before and after the interventions to allow us to measure the efficacy of the intervention. The interventions used were posters to remind nursing staff to document bowel movements on the electronic patient chart using the Bristol Stool Chart. Staff were advised to include bowel movement documentation as a standard step in the handover checklist between shifts and to include it in the daily nursing care bundles.

Results

After implementing the interventions, significant improvements were seen in bowel documentation. Daily bowel movement documentation increased from 27% to 73%, and Bristol Stool Chart use improved from 57% to 87%. Stool frequency documentation doubled from 30% to 60%. Clinical actions decreased slightly, from 45% to 39%. Correct chart format use improved from 47% to 87%, and stool consistency documentation rose from 50% to 87%, indicating enhanced care and monitoring.

Conclusion

Overall, simple interventions resulted in significant improvement in documentation. Consistent documentation enables early intervention and helps prevent patient deterioration, as well as delays in discharge.

## Introduction

Bowel habits are a crucial indicator of a person’s overall well-being. They can become disturbed when a person is out of their natural environment. In the hospital setting, several factors influence patients’ bowel movements, including changes in diet and fluid intake, reduced mobility, certain medications, psychological stress, and disruptions to their everyday routine.

The Bristol Stool Chart is a diagnostic medical tool used to classify faeces into seven categories [1]. It is easy to use, commonly available in most hospital settings, and often displayed on walls around the ward.

In the NHS, it is often the nursing staff’s responsibility to document the amount, consistency, type, and frequency of bowel movements using the Bristol Stool Chart. They should also escalate if any abnormal bowel movements (types 1, 2, 6, or 7) occur and raise concern if the stool has an abnormal colour.

Ward doctors are responsible for reviewing nursing documentation and taking appropriate action. On several occasions, patients complain of constipation or diarrhoea, but when medical records are checked, there is a lack of documentation. This often results in delays in initiating treatment or discharge. It also leads to more radiological imaging being performed, and patients may require surgical intervention due to suspicion of obstruction.

Elderly patients are most often affected by a lack of documentation and are particularly affected by constipation. The prevalence rises to between 30% and 40% in those aged 65 and above [2]. Constipation in the elderly can lead to confusion and, in severe cases, urinary retention and bowel obstruction. Although it may seem like a trivial matter, it has a significant impact on quality of life and psychological well-being [3].

The primary aim of this Quality Improvement Project (QIP) is to improve the accuracy and consistency of bowel documentation using the Bristol Stool Chart, improve patient care through early intervention, and reduce the financial burden on the NHS by limiting radiological investigations.

## Materials and methods

This is a retrospective study, and the population consisted of patients from two Diabetes and Endocrine wards in a District General Hospital (DGH) in Blackburn, United Kingdom. The study took place from April to October 2025. Approval was obtained from the designated appraisal team at the hospital, and they were regularly updated on data collection and the completion of each cycle. Formal ethical approval was not required for the patients included in the study, as there was no direct involvement.

The QIP was designed using a Plan Do Study Act cycle (PDSA). The plan was to improve bowel movement documentation.

Thirty patients were selected: fifteen from ward D1 (Endocrine and Diabetes ward) and fifteen from ward D3. A systematic, ward-based sampling approach was employed. Data collection began with the patient occupying Bed 1 in each of the two study wards. The electronic record of the patient in that bed at the time of the audit was reviewed. If the patient met all inclusion criteria, they were included in the study. If the patient did not meet the criteria, the next consecutive bed was assessed. This process continued sequentially through the wards until a total of 30 eligible patients was obtained for each cycle. This method ensured consecutive, unbiased sampling without preferential selection based on clinical condition or documentation quality.

The inclusion criteria included all patients, regardless of age, gender, or ethnicity, who had an admission date more than three days prior. The study excluded patients who were on the end-of-life (EOL) pathway and those with incomplete documentation due to transfer or early discharge (Table 1).

**Table 1:** Inclusion and exclusion criteria.

Inclusion Criteria	Exclusion Criteria
• Length of stay > 3 days	• Patients on the End-of-Life (EOL) pathway
• Complete electronic documentation available for the admission period	• Incomplete documentation due to early discharge
• No restrictions on age, sex, or ethnicity	• Incomplete documentation due to transfer to another ward

The first cycle included reviewing data from 30 patients in total from the electronic records. Six categories were assessed: bowel movement documented daily, recorded using the Bristol Stool Chart, stool frequency, clinical action taken, correct chart format used, and consistency. The data was analysed using Microsoft Excel.

All the data was collected retrospectively. For each patient, electronic records were reviewed from the start of their admission to the ward, and all relevant documentation was examined from the point of admission up to the day of data collection.

Following this, a questionnaire was administered to 12 nursing staff members who were caring for these patients for root cause analysis (Appendix 1).

Data revealed that the most common causes related to documentation practice were time constraints and forgetfulness.

**Figure 1:**
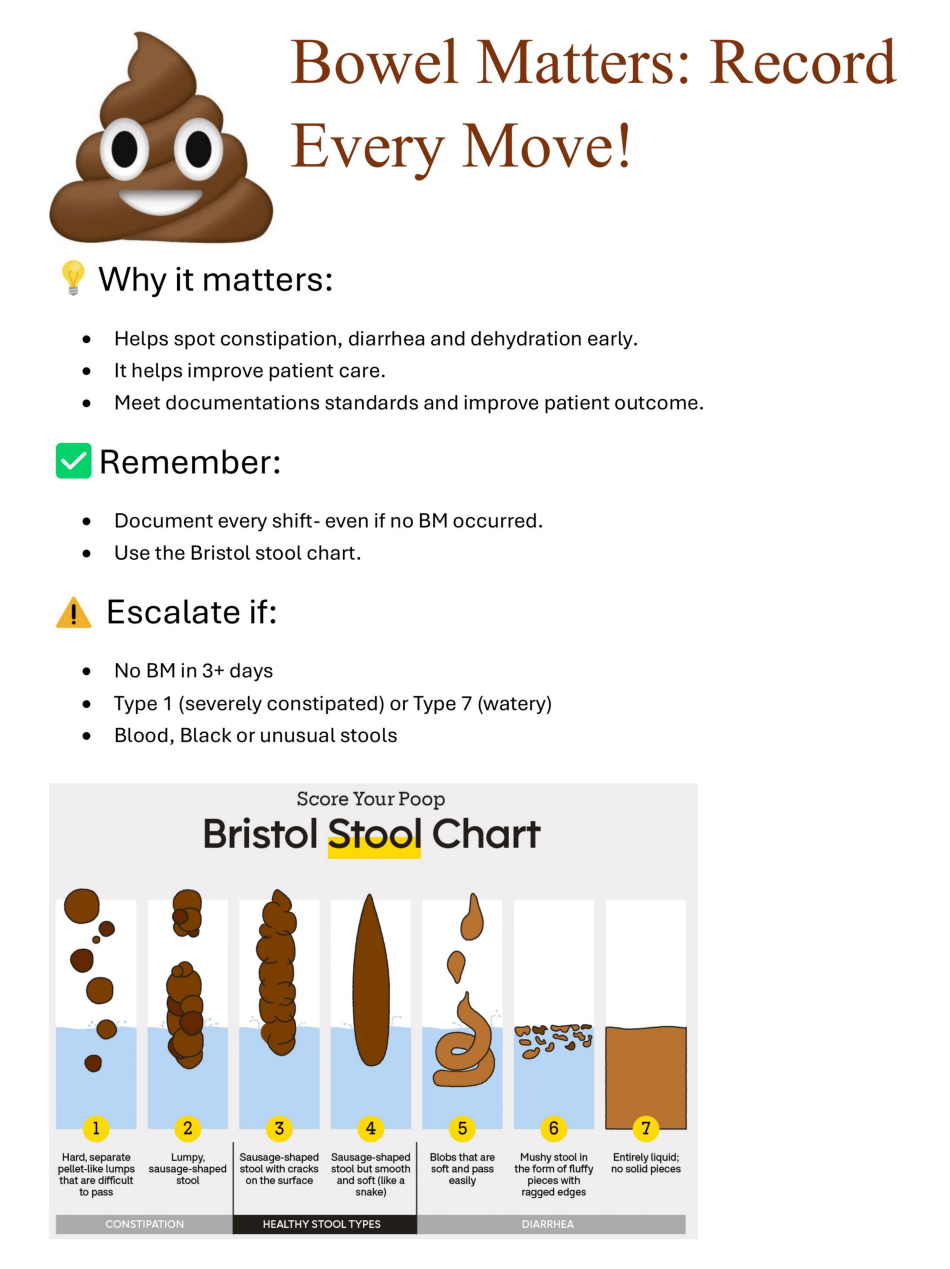
Poster: “Bowel Matters,” a reminder for staff to document bowel movements. Poster designed by Sahar Yaseen.

To address forgetfulness, poster reminders (Figure 1) were placed throughout the ward in areas where nursing staff spent most of their time. Posters were also placed at the staff workstation and in in-patient toilets. The ward manager was provided with a summary of the information on the poster and was advised to communicate it to the rest of the team members involved in the patient’s care.

The second cycle of data was collected one month later, using the same study design, and the same analysis was performed using Microsoft Excel.

The study was initially conducted in the Diabetes and Endocrine wards. Following the completion of two audit cycles and after observing the improvement that the interventions had brought, the changes were implemented in other wards, including the medicine for older people (MFOP) ward, the rehabilitation wards, and the stroke wards. The changes were discussed in in-person departmental teaching sessions and multidisciplinary team meetings.

## Results

The QIP aimed to enhance patient-centred care by improving the accuracy and consistency of bowel documentation.

**Figure 2:**
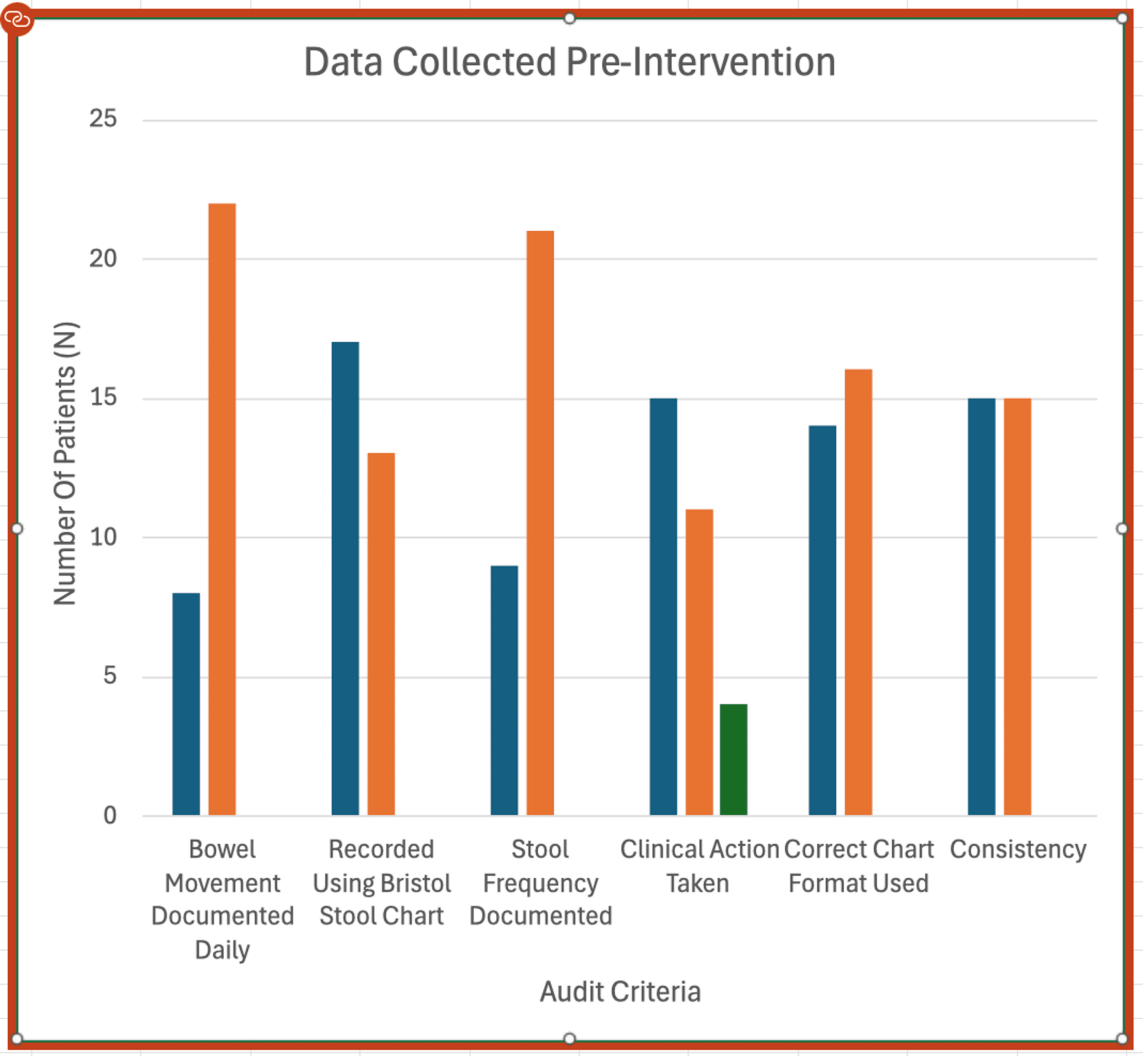
Data collected pre-intervention from wards D1 and D3. Data collected pre-intervention from wards D1 and D3 are presented in a column chart. Six documentation categories were measured against the number of patients (N).

**Figure 3:**
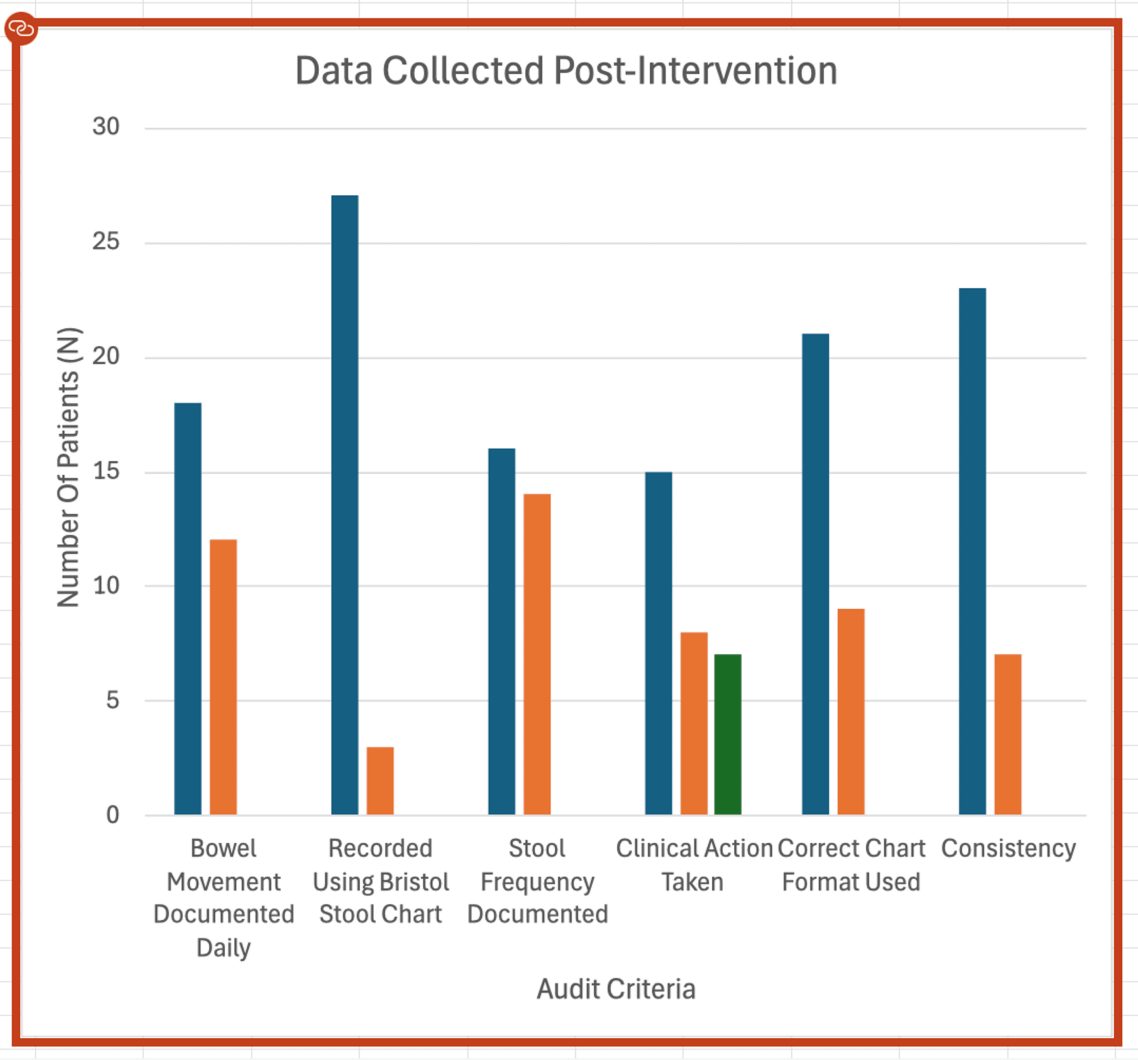
Data collected post-intervention from wards D1 and D3. Data collected post-intervention from wards D1 and D3 are presented in a column chart. Six documentation categories were measured against the number of patients (N).

The results from the second audit cycle show a marked improvement in bowel documentation practices across all key areas following the implementation of targeted interventions, as demonstrated in Figures 2-3.

Daily bowel movement documentation improved markedly, increasing from 8 cases (27%) in cycle one (Figure 2) to 18 cases (73%) in cycle two (re-audit), indicating better routine monitoring by staff (Figure 3).

Use of the Bristol Stool Chart rose from 17 cases (57%) to 27 cases (87%), reflecting greater adherence to standardised documentation practices (Figure 3).

Stool frequency documentation increased from 9 cases (30%) to 16 cases (60%), highlighting enhanced attention to patient bowel habits (Figures 2-3).

In cycle one, clinical action was taken in fifteen cases (50%), not taken in eleven cases (37%), and deemed unnecessary in four cases (13%).

In cycle two, clinical action was taken in fifteen cases (50%), not taken in eight cases (27%), and not required in seven cases (23%). The data suggest that accurate documentation enabled staff to more accurately determine when clinical intervention was necessary (Tables 2-3).

**Table 2:** Data collected from Audit 1.

Category	Yes (N, %)	No (N, %)	Not Required (N, %)
Bowel movement documented daily	8 (27%)	22 (73%)	-
Recorded using Bristol Stool Chart	17 (57%)	13 (43%)	-
Stool frequency documented	9 (30%)	21 (70%)	-
Clinical action taken	15 (50%)	11 (37%)	4 (13%)
Correct chart format used	14 (47%)	16 (53%)	-
Consistency	15 (50%)	15 (50%)	-

**Table 3:** Data collected from Audit 2.

Category	Yes (N, %)	No (N, %)	Not Required (N, %)
Bowel movement documented daily	18 (73%)	12 (27%)	-
Recorded using Bristol Stool Chart	27 (87%)	3 (13%)	-
Stool frequency documented	16 (60%)	14 (40%)	-
Clinical action taken	15 (50%)	8 (27%)	7 (23%)
Correct chart format used	21 (70%)	9 (30%)	-
Consistency	23 (87%)	7 (13%)	-

The decrease in clinical actions can be explained by the fact that accurate bowel documentation enabled staff to recognise trends earlier and intervene promptly with timely and less invasive measures, such as adjustments to diet and fluid intake, to support bowel movement.

Use of the correct chart format improved from 14 cases (47%) to 21 cases (87%), showing greater consistency in using the appropriate documentation tools (Figures 2-3).

Stool consistency documentation also increased significantly, rising from 15 cases (50%) to 23 cases (87%), indicating more thorough and detailed recording.

These improvements suggest that the implemented interventions, including staff education, poster summaries, and engagement with ward managers, were associated with improved bowel documentation practices. Enhanced documentation supports safer, more patient-centred care by reducing the risk of missed diagnoses and unnecessary investigations, which carry significant cost implications for the NHS. As the outcomes measured were categorical variables (Yes/No/Not Required) and involved independent observations across two audit cycles, the chi-square test was an appropriate statistical method to compare proportions before and after the interventions. Statistical analysis demonstrated statistically significant improvements in most documentation categories (p < 0.05; Table 4), supporting a meaningful improvement in documentation practices following the interventions.

**Table 4:** Statistical association between documentation and clinical outcome. Chi-square (χ²) tests were conducted for each of the six categories. A p-value < 0.05 was considered statistically significant, indicating a meaningful relationship between documentation quality and reduced clinical interventions.

Category	χ²(1)	p-value
Bowel movement documented daily	6.787	0.0092
Bristol Stool Chart used	8.523	0.0035
Stool frequency documented	3.36	0.0668
Clinical action taken	8.673	0.0032
Correct chart format used	3.36	0.0668
Consistency documented	4.593	0.0321

## Discussion

The results clearly demonstrate how a small intervention can have a significant impact on patient care. More accurate and consistent bowel documentation is also a better indicator of effective communication between the team members involved in the patient's care.

Changes in bowel movements are often not communicated by patients themselves due to several limiting factors; hence, it's essential that these changes are documented so that they can be addressed by the doctor and actioned accordingly.

Constipation is a distressing condition that leads to pain, discomfort, nausea, reduced appetite, and even severe conditions like bowel obstruction or perforation [3]. On the other hand, type 7 stools can indicate dehydration. If type 7 occurs three times or more in a day, it can be an indication of *Clostridium difficile* infection, requiring a person to be isolated and certain prevention methods to be implemented, such as hand washing with soap and water, contact precautions, and thorough cleaning [4].

The elderly are more prone to deterioration from constipation. This is particularly true for elderly patients who have cognitive impairment or dementia, as it can be challenging for them to express their symptoms; they may instead present with non-specific symptoms such as aggression [5].

One of the strengths of this QIP is that it can be implemented on all internal medicine wards. It is simple and cost-effective. The Bristol Stool Chart is a widely recognised tool that most staff working in the NHS are aware of from their early education or training years. Small interventions, such as posters and brief educational teaching sessions during multidisciplinary team (MDT) meetings, have led to a positive outcome in patient care. The visual pictures on the poster are easy to understand for staff with varied clinical backgrounds, including newly qualified nurses, health care assistants, and agency staff. These findings are consistent with existing literature, which supports the use of visual aids in improving clinical communication [6]. Another strength of this project is that it aligned with existing practices for some team members, meaning it did not require extensive training or significant changes to their daily routine.

Despite the positive results, this QIP has some limitations. A key limitation of this project is the potential for short-term bias, given that the interventions were newly introduced and ward staff were initially more motivated to adopt the new documentation practice; therefore, sustainability may be limited over time. Additionally, the observed improvement could be partially attributed to the Hawthorne effect, where staff improve documentation due to being monitored or fear of being questioned about undocumented practices [7], although this effect was not directly measured. The sample size is also a limitation, as it involved a very small cohort of 30 patients per cycle, which reduces the validity of the findings. Another limitation is that the follow-up period was short, which restricts the ability to assess whether the improvements would be sustained in the long term. Furthermore, this was a single-centre study conducted in only two wards within one specialty, which limits the generalisability of the findings to other wards or hospital settings where staffing workflows and documentation practices may differ.

For further improvements, this project could focus on sustainable strategies such as implementing electronic reminders and conducting regular refresher sessions. Frequent teaching sessions can address new or ongoing concerns and may help reduce the Hawthorne effect by shifting staff motivation from performing because they are observed to genuine engagement. Additionally, recognising staff efforts through verbal praise or feedback can reinforce positive behaviour, encourage adherence, and help sustain improvements over time, ensuring that changes persist beyond the initial monitoring period.

Another valuable step could be to conduct a further audit to assess the clinical outcomes of consistent and accurate bowel documentation.

## Conclusions

Overall, there has been a significant improvement, which is reflected in the quality of patient care. The main aim of the QIP has been achieved; however, there remains considerable scope for further improvement.

Consistent and accurate bowel documentation is a crucial aspect of providing patients with good-quality care and maintaining dignity. Early intervention can prevent increased morbidity and mortality and may also reduce the risk of hospital-acquired infections.

Additionally, psychological well-being is closely linked to bowel health. Symptoms such as constipation or diarrhoea can significantly affect mental health, while psychological distress can, in turn, impact bowel function. Addressing this bidirectional relationship is crucial in delivering comprehensive patient care.

## Appendices

Appendix 1 

**Table 5:** Staff questionnaire on bowel movement documentation practices. This questionnaire was given to 12 nursing staff members on both wards to complete anonymously.

Question No.	Question	Response Format	Section
1	Are you familiar with the Bristol Stool Chart?	Yes / No / Not sure	Awareness and Knowledge
2	Have you received training on how to use the Bristol Stool Chart?	Yes / No	Awareness and Knowledge
3	Do you know where to document bowel movements in Cerner?	Yes / No	Awareness and Knowledge
4	How often do you document bowel movements for your patients?	Always / Often / Sometimes / Rarely / Never	Documentation Practices
5	What factors influence whether you document a patient’s bowel movement?	Time constraints; Clinical relevance at the time; Forgetfulness; Unclear documentation protocols; Lack of awareness; System (e.g., electronic records) is difficult to navigate; Other (please specify): (Short answer)	Documentation Practices
6	Do you feel confident in assessing and documenting stool characteristics (e.g., consistency, frequency, appearance)?	Yes / Somewhat / No	Documentation Practices
7	What are the main reasons you may not document a bowel movement?	Open-ended (paragraph)	Barriers and Attitudes
8	Do you believe bowel movement documentation is clinically important? Why or why not?	Open-ended (paragraph)	Barriers and Attitudes
9	Would more training/teaching or clearer guidelines help improve your documentation of bowel movements?	Yes / No / Not sure	Barriers and Attitudes
10	Do you feel that the correct clinical actions are being taken when a patient has bowel-related issues (e.g., constipation, diarrhoea, abnormal stool)?	Yes / Sometimes / No	Barriers and Attitudes
